# Ir impurities in $\alpha$- and $\beta$-$\text {Ga}_{2}\text {O}_{3}$ and their detrimental effect on p-type conductivity

**DOI:** 10.1038/s41598-023-35112-9

**Published:** 2023-05-26

**Authors:** Aleksandrs Zachinskis, Jurij Grechenkov, Edgars Butanovs, Aleksandrs Platonenko, Sergei Piskunov, Anatoli I. Popov, Juris Purans, Dmitry Bocharov

**Affiliations:** 1 Institute of Solid State Physics, University of Latvia, 8 Kengaraga str., Riga, LV-1063 Latvia; 2 Transport and Telecommunication Institute, 1 Lomonosova str., Riga, LV-1019 Latvia

**Keywords:** Condensed-matter physics, Materials science, Electronic structure, Semiconductors, Electronic properties and materials

## Abstract

Recently gallium oxide ($\text {Ga}_{2}\text {O}_{3}$) has become one of the most actively studied materials due to its competitive electronic properties such as wide bandgap, high breakdown field, simple control of carrier concentration, and high thermal stability. These properties make gallium oxide a promising candidate for potential applications in high-power electronic devices. $\beta$-$\text {Ga}_{2}\text {O}_{3}$ crystals are commonly grown by the Czochralski method in an iridium (Ir) crucible. For this reason, Ir is often present in $\text {Ga}_{2}\text {O}_{3}$ crystals as an unintentional dopant. In this work the impact of Ir incorporation defects on potential p-type conductivity in $\beta$-$\text {Ga}_{2}\text {O}_{3}$ is studied by means of density functional theory. The metastable $\alpha$-$\text {Ga}_{2}\text {O}_{3}$ phase was investigated as the model object to understand the processes caused by iridium doping in gallium oxide-based systems. Obtained results allow us to understand better the influence of Ir on $\text {Ga}_{2}\text {O}_{3}$ electronic structure, as well as provide interpretation for optical transitions reported in recent experiments.

## Introduction

Gallium oxide in its beta-phase ($\beta$-$\hbox {Ga}_{2}\hbox {O}_{3}$) is a wide bandgap (4.7–4.9 eV^1,2^) semiconductor that has recently drawn significant attention becoming one of the most actively studied materials. Its promising properties such as a wide bandgap, high breakdown field (8 MV/cm), and high thermal and chemical stability make $\beta$-$\hbox {Ga}_{2}\hbox {O}_{3}$ being a strong candidate for applications in high-power electronics^3,4^, such as Schottky diodes^5,6^ and field-effect transistors^7^, as well as in Boltzmann thermometers^8^, solar-blind ultraviolet (UV) photodetectors^4^, scintillators^9^, and others^10^. Monoclinic $\beta$-$\hbox {Ga}_{2}\hbox {O}_{3}$ is thermodynamically the most stable crystalline phase^11^. A metastable hexagonal $\alpha$-$\hbox {Ga}_{2}\hbox {O}_{3}$ is structurally similar to the corundum and has a slightly wider band gap of 5.1–5.3 eV^12,13^. This phase can be obtained using various thin film deposition processes (*e*.*g*. radio frequency (RF) sputtering, halide vapor phase epitaxy (HVPE), molecular beam epitaxy (MBE), atomic layer deposition (ALD), mist chemical vapor deposition (mist-CVD))^14^, and could overcome $\beta$-$\hbox {Ga}_{2}\hbox {O}_{3}$ in device performance^15^.

Heterojunctions are necessary for many device applications, thus the control of n- and p-type conductivity is important. N-type doping is easily achievable through addition of Si, Sn, C and Ge impurities^16–18^. Oxygen vacancies, which are native defects, can also act as electron donors^19^. Nb doping has been suggested to achieve similar effect elsewhere^20^. While n-type $\hbox {Ga}_{2}\hbox {O}_{3}$ has been successfully synthesized, p-type doping remains yet a challenge^21^. Most promising candidates for p-type doping are Mg and N, although induced defects levels are relatively deep^17^. Ismam et al.^22^ discuss the employment of H-interstitials to control both p- and n-nype conductivity, nevertheless the hole mobility is rather low. Theoretical studies propose N–P, Al–N, and In–N co-doping to obtain a conductivity of p-type^23,24^. Nonetheless, challenges remain plenty: oxygen vacancies tend to counteract the proposed acceptors, even though this may be solved by annealing in O-rich atmosphere, Mg defects and gallium vacancies that also act as acceptors are passivated by hydrogen^19,25^, and thus holes become self-trapped near an oxygen atom^26^.

$\beta$-$\hbox {Ga}_{2}\hbox {O}_{3}$ crystals are grown by Czochralski (CZ) method using iridium (Ir) crucible^27,28^. As a result, iridium is present in $\beta$-$\hbox {Ga}_{2}\hbox {O}_{3}$ as an unintentional dopant^27^ and it is speculated that Ir dopant may affect the p-type conductivity^25^. In n-type $\beta$-$\hbox {Ga}_{2}\hbox {O}_{3}$ Ir is in Ir$^{3+}$ charged state^17^. Ir$^{4+}$ charged state is also possible at low enough Fermi level, which can be achieved by introducing Mg impurities^17,29^. According to calculations reported by Ritter et al. ^25^, Ir incorporates in octahedral $\hbox {Ga}_\text {II}$ site. In the octahedral crystal field 5 *d* orbitals of Ir split into 3 $t_{2g}$ low energy orbitals and 2 $e_g$ orbitals with higher energy. Ir$^{3+}$ ($5d^6$) has no electron paramagnetic resonance (EPR) signal because six *d* electrons occupy 3 $t_{2g}$ orbitals ($\uparrow \downarrow \ \uparrow \downarrow \ \uparrow \downarrow$)^30^. Ir$^{4+}$ ($5d^5$), on the other hand, has a spin state S=1/2 ($\uparrow \downarrow \ \uparrow \downarrow \ \uparrow$)^30^.

In Mg-doped samples IR absorption peak at around 5150 cm$^{-1}$ was reported in several experiments^25,30–32^. Origin of the peak is attributed to Ir$^{4+}$ impurity, specifically, to a *d*-*d* transition within $t_{2g}$ orbitals. Additionally, Seyidov et al.^32^ observed that the intensity of the 5150 cm$^{-1}$ peak reached its maximum at optical excitation of 2.9 eV in ERS (Electronic Raman Scattering) experiment. The maximum of 2.9 eV was hypothetically assigned to transition from $t_{2g}$ orbitals to $e_g$ orbitals located in the conduction band.

In this study we investigated electronic properties of Ir doped $\beta$-$\hbox {Ga}_{2}\hbox {O}_{3}$ by means of Density Functional Theory (DFT) calculations implemented via CRYSTAL17 code^33,34^, assessed the impact of iridium impurities on the possibility of p-type doping in this material as well as evaluated the undesirable effects associated with the presence of iridium impurities. The calculated formation energies, charge-state transition levels, and electronic band structure are compared with available experimental results. Additionally, we investigated the role of Ir impurities in $\alpha$-$\hbox {Ga}_{2}\hbox {O}_{3}$ phase. In these calculations $\alpha$-$\hbox {Ga}_{2}\hbox {O}_{3}$ was used as a model object for which the effect of iridium doping was evaluated, as well as the similarities and differences with the stable $\beta$-$\hbox {Ga}_{2}\hbox {O}_{3}$, in which iridium impurities occur during actual material synthesis, were analyzed in order to predict the processes that may occur, for example, in samples with a mixed phase content.

## Results

Calculated equilibrium lattice constants of pure $\alpha$-$\hbox {Ga}_{2}\hbox {O}_{3}$ and $\beta$-$\hbox {Ga}_{2}\hbox {O}_{3}$ showed excellent agreement with experimental data (see Table 1). The calculated indirect bandgap of 4.73 eV for $\beta$-$\hbox {Ga}_{2}\hbox {O}_{3}$ and 5.29 eV for $\alpha$-$\hbox {Ga}_{2}\hbox {O}_{3}$ are also in agreement with those experimentally measured (see Table 1). Direct bandgap is about 0.05 eV wider and is equal to 4.78 eV for $\beta$-$\hbox {Ga}_{2}\hbox {O}_{3}$ at k-point $\Gamma$, while for $\alpha$-$\hbox {Ga}_{2}\hbox {O}_{3}$ direct bandgap is 5.52 eV, which is 0.23 eV wider than indirect, also at $\Gamma$.

**Table 1:** Lattice constants and bandgap energy of pure $\alpha$-$\hbox {Ga}_{2}\hbox {O}_{3}$ and $\beta$-$\hbox {Ga}_{2}\hbox {O}_{3}$ phases calculated with hybrid functional HSE06 and double-zeta basis set. Experimental data is given for reference.

Phase	Parameter	This work	Exp.
$\beta$	a, Å	12.261	12.214^35^
	b, Å	3.0436	3.0371^35^
	c, Å	5.8164	5.7981^35^
	$\beta$	103.83$^o$	103.83$^o$^35^
	bandgap, eV	4.73	4.7^1^
$\alpha$	a, Å	4.9953	4.9825^36^
	c, Å	13.408	13.433^36^
	bandgap, eV	5.29	5.27-5.3^12,37,38^

**Figure 1:**
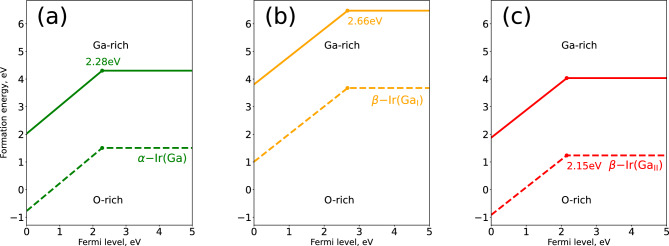
Formation energy of Ir substitution defects in both $\alpha$-$\hbox {Ga}_{2}\hbox {O}_{3}$ (**a**) and $\beta$-$\hbox {Ga}_{2}\hbox {O}_{3}$ ((**b**) for Ir atom in Ga$_\text {I}$ and (**c**) for Ir atom in Ga$_\text {II}$ two nonequivalent position, correspondingly) and as calculated according to Eq. (2). Solid and dashed lines correspond to Ga-rich and O-rich conditions, respectively.

The calculated formation energies of iridium defects are shown in Fig. 1. Most favorable substitution site is octahedral Ga$_\text {II}$ in $\beta$-$\hbox {Ga}_{2}\hbox {O}_{3}$, consistent with reported by Ritter et al.^25^. Assuming bandgap of 4.73 eV, calculated thermodynamic charge state transition level $\varepsilon (+/0)$ of Ir in Ga$_\text {II}$ site in $\beta$-$\hbox {Ga}_{2}\hbox {O}_{3}$ is located 2.6 eV below the conduction band minimum (CBM). Value of 2.6 eV below CBM is in good agreement with that experimentally measured 2.2-2.3 eV below CBM^31^.

Iridium in $\beta$-$\hbox {Ga}_{2}\hbox {O}_{3}$ was observed in Ir$^{3+}$ (5$d^6$) and Ir$^{4+}$ (5$d^5$) states^30^. Under the influence of a strong crystal field, *d*-orbitals split. According to our calculations, the ground state of Ir$^{3+}$ in both nonequivalent sites is non-magnetic (S=0) ($\uparrow \downarrow \ \uparrow \downarrow \ \uparrow \downarrow$). On the other hand, Ir$^{4+}$ in octahedral Ir$_{\hbox {Ga}_{\text {II}}}$ and tetrahedral Ir$_{\hbox {Ga}_{\text {I}}}$ sites converge to spin state S = 1/2 ($\uparrow \downarrow \ \uparrow \downarrow \ \uparrow$). This result is in agreement with EPR experiment^30^. We investigated spin states further, and found that for Ir$^{3+}_{\hbox {Ga}_{\text {I}}}$ spin states S=2/2 and S=4/2 are 0.3 eV and 1.1 eV more favorable than S=0, respectively. The spin states of both Ir$^{3+}_{\hbox {Ga}_{\text {II}}}$ and Ir$^{3+}_{\hbox {Ga}}$ (in $\alpha$-$\hbox {Ga}_{2}\hbox {O}_{3}$) also converged to S=4/2. They are 3.8 eV more favorable with respect to S=0. Ir$^{4+}_{\hbox {Ga}_{\text {I}}}$ also converged to S=3/2, which is 0.9 eV more favorable than S=1/2. High-spin configuration S=5/2 is not predicted for Ir$^{4+}$ substitution dopant.

**Figure 2:**
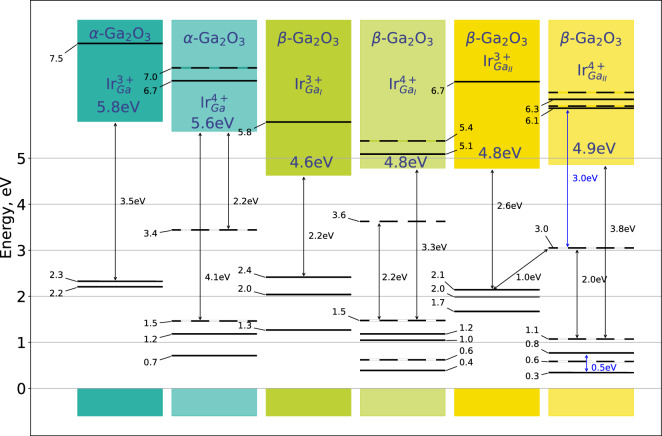
Schematic illustration of band structure of $\alpha$-$\hbox {Ga}_{2}\hbox {O}_{3}$ and $\beta$-$\hbox {Ga}_{2}\hbox {O}_{3}$ with Ir impurities. Solid and dashed lines distinguish between alpha and beta electrons. All energy levels were evaluated at k-point $\Gamma$. Energy levels of Ir in the conduction band were also included. All valence band maxima were aligned and set to zero energy.

Kohn-Sham levels of Ir for both $\alpha$-$\hbox {Ga}_{2}\hbox {O}_{3}$ and $\beta$-$\hbox {Ga}_{2}\hbox {O}_{3}$ from band structure calculations are illustrated in Fig. 2. Energy levels located in the conduction band were also added. IR peak at 5150 cm$^{-1}$ ( 0.64 eV) was observed in several experiments and attributed to a transition within $t_2g$ orbitals in iridium atom substituting octahedral $\hbox {Ga}_\text {II}$ in $\beta$-$\hbox {Ga}_{2}\hbox {O}_{3}$. The intensity of 5150 cm$^{-1}$ peak was at a maximum when the sample was irradiated with 2.9 eV light^32^. The energy of 2.9 eV was hypothetically attributed to electronic transition in Ir$^{4+}$(Ga$_\text {II}$) between $t_{2g}$ and $e_g$ orbitals in $\beta$-$\hbox {Ga}_{2}\hbox {O}_{3}$, assuming $e_g$ orbitals are located in conduction band^32^.

From the calculated band structure we see a possible electronic transition in Ir$^{4+}$(Ga$_\text {II}$) (last column) at the energy of 3.0 eV in $\beta$-$\hbox {Ga}_{2}\hbox {O}_{3}$ that most likely correspond to 2.9 eV transition reported by Seyidov et.al.^32^. Furthermore, we see two possible transitions with the energy of around 0.5 eV ($\approx$4000 cm$^{-1}$) that most likely correspond to 5150 cm$^{-1}$ peak. One can further conclude that the transitions of the defect from a Ir$^{3+}$ to a Ir$^{4+}$ state at a certain Fermi level leads to Ir acting as a hole trap.

In other words iridium defects inhibit the p-type conductivity in the material after the hole concentration reaches a certain level. This concentration can be estimated by the use of the mass action law^39^:

1 $$p = N_{v} e^{{\left( { - \frac{{E_{F} - E_{V} }}{{k_{B} T}}} \right)}}$$

where *p* is hole concentration, $E_F$—Fermi level $E_V$—valence band energy, and $N_v$—hole concentration at valence band edge.

From our computations $E_V-E_{F,\,critical} \approx 2.7 \; eV$ and the number of states in a $\approx 25 \; \text{meV}$ close to the valence band edge is $N_V \approx 0.11$ per unit cell. Assuming room temperature ($T=300\;\text{K}$), one can obtain a noticeably low hole concentration of $p \approx 3 \times 10^{-15} \; \text{cm}^{-3}$ that should be approximately equal to the dopant concentration, for which the effect takes place.

Additionally, from Fig. 2 we can conclude following statements about Ir dopants $\beta$-$\hbox {Ga}_{2}\hbox {O}_{3}$:

1. The indirect bang gap of 4.78 eV in $\beta$-$\hbox {Ga}_{2}\hbox {O}_{3}$ practically does not change when iridium impurities are introduced.
2. The position of the levels in the band gap depends on the Ga-ion position in which it is located. Moreover, this is true for both charge states of iridium, namely, Ir$^{3+}$ and Ir$^{4+}$.
3. In the case of Ir$^{3+}$, the position of its ground state with respect to the bottom of the conduction band is 2.2 eV and 2.6 eV for Ga$_\text {I}$ site and Ga$_\text {II}$ site, respectively. These energies formally correspond to the ionization energy ${Ir}^{3+}\rightarrow {Ir}^{4+}$, this difference should be easily distinguishable in the experiment. Note that the threshold for exciting an electron from the Ir$^{3+}$ level to the conduction band minimum was found to be between 2.2 and 2.3 eV^31^, which corresponds to the position of iridium in Ga$_\text {I}$.
4. Strange as it may seem, but only for Ga$_\text {I}$ position our calculations show the presence of an excited quasi-local state ${Ir}^{3+}$, which is higher than the bottom of CB by about 1 eV. Furthermore, we can assume the presence of the optical absorption band of Ir$^{3+}$ at 3.2 eV.
5. The presence of an unoccupied level of Ir$^{3+}$(Ga$_\text {I}$) in the conduction band hints to a possibility of it being a center of an electron capture with the formation of transient states of Ir$^{2+}$, according to the following reaction: Ir$^{3+}$ + e $\rightarrow$ Ir$^{2+}$. Note that a summary of Ir$^{2+}$ ions in the different compound was done by Pidol^40^ *et.al.*.

**Figure 3:**
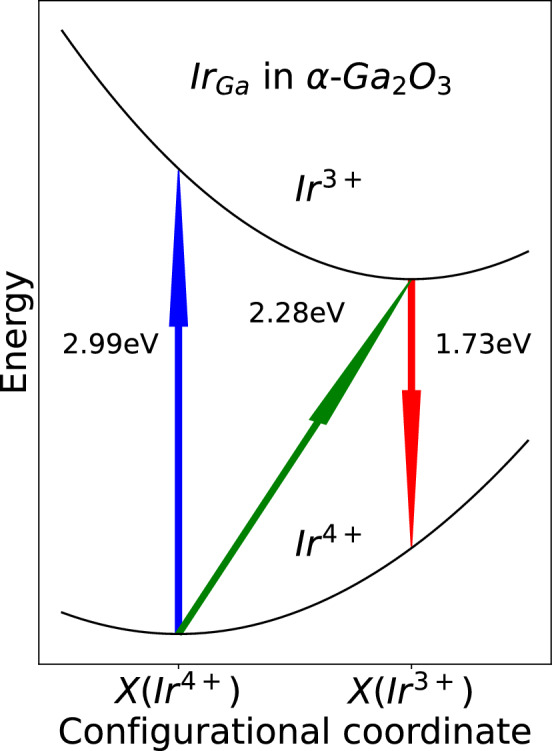
Configurational diagram of optical transition levels of Ir$_{\hbox {Ga}_{\text {II}}}$ in $\beta$-$\hbox {Ga}_{2}\hbox {O}_{3}$ and Ir$_{\hbox {Ga}}$ in $\alpha$-$\hbox {Ga}_{2}\hbox {O}_{3}$, exchanging an electron with valence band. $X({Ir}^{4+})$ and $X({Ir}^{3+})$ corresponds to the geometry of the ground state of Ir$^{4+}$ and Ir$^{3+}$, respectively.

Following the work of Ritter et al.^25^ we calculated also optical transition levels of Ir$_{\hbox {Ga}_{\text {II}}}$ in $\beta$-$\hbox {Ga}_{2}\hbox {O}_{3}$ and Ir$_{\hbox {Ga}}$ in $\alpha$-$\hbox {Ga}_{2}\hbox {O}_{3}$. The difference between thermodynamic and optical charge state transition levels is that no relaxation of geometry is performed. Results are illustrated in the Fig. 3. Absorption and emission energy (blue and red arrow, respectively) differs by about 0.6 eV from thermodynamics transition for both $\alpha$ and $\beta$ phases.

## Methods

### DFT calculations

DFT calculations were performed using linear combination of atomic orbitals (LCAO) method and HSE06 hybrid exchange-correlation functional^41^. In the DFT method, the energy of a system is expressed as a functional of the spatial electron density. Hybrid functionals, like HSE06, are designed to overcome some of the limitations of traditional DFT functionals by incorporating a certain amount of exact exchange energy. The screening parameter in HSE06 determines the range separation between short- and long-range interactions in the functional. This screened Coulomb potential weakens the long-range contribution of the exact exchange mitigating the self-interaction error frequently observed in standard DFT functionals. The HSE06 functional incorporates a 25 % short-range Hartree-Fock exchange without any long-range Hartree-Fock exchange. In the most recent version of the functional, the screening parameter is chosen empirically and assigned a value of 0.11 Bohr$^{-1}$.^42^ Gaussian type double-zeta basis sets developed by Vilela Oliveira et al.^43^ were used for O and Ga atoms. A double-zeta basis set in LCAO method employs two basis functions for each atomic orbital instead of just one, as in a single-zeta basis set. This means that for each AO in the atom, there are two functions with different radial extents (contraction coefficients) and adjustable weights. The purpose of using two functions is to provide a more flexible representation of the electron distribution around the nucleus, thereby improving the description of molecular orbitals and the overall accuracy of the calculations. For the Ir atom an effective core triple-zeta split-valence plus polarization pseudopotential basis set was taken in the form as it was suggested by Pint et al.^44^ and Chesnokov et al.^45^.

The supercell approach was adopted to model isolated Ir defect. A 120 atom 1$\times$3$\times$2 supercell (relative to crystallographic cell) was used for $\beta$-$\hbox {Ga}_{2}\hbox {O}_{3}$. The same 120 atom size 2$\times$2$\times$1 supercell was utilized for $\alpha$-$\hbox {Ga}_{2}\hbox {O}_{3}$ calculations. 4$\times$4$\times$4 Pack-Monkhorst net was used, resulting in 36 k-points in the first Brillouin zone. The threshold for the energy convergence in self-consistent field (SCF) procedure was set to be 10$^{-7}$ Hartree. Complete relaxation of atomic coordinates has been performed. Other computational parameters were left as default (see CRYSTAL17 manual^33^).

Formation energies of Ir defects were calculated using the relation:

2 $$\begin{aligned} E^f = E[X^q] - E[I] - \sum _{i} n_i \mu _i + q E_F + E_{\text {corr}} \end{aligned}$$

$E[X^q]$ is the total energy of a defective system with a charge *q*, *E*[*I*] is the total energy of an ideal system, $n_i$ is a number of added atoms with chemical potential $\mu _i$ (if an atom was removed, then $n_i$ is negative), $E_F$ is the Fermi level relative to valence band maximum (VBM) and $E_{\text {corr}}$ is the correction energy due to electrostatic interactions of charged defects. For the charged Ir$^{4+}$ ions, correction energy $E_{\text {corr}}$ for first order electrostatic interactions is calculated following the Makov-Payne correction scheme^46^. We follow the methodology of computing correction energy in the CRYSTAL code as is described by Bailey et al. ^47^. For more details, see the next subsection.

Since chemical potentials may vary depending on experimental conditions we considered two limiting scenarios, namely, O-rich and Ga-rich (O-poor) conditions. In the O-rich condition, the system is assumed to have a high concentration of oxygen compared to gallium. The chemical potential of oxygen is then calculated based on the total energy of an oxygen molecule, *i*.*e*. half of the total energy of an $\hbox {O}_{2}$ was taken as $\mu$ for O. The $\mu _{\textrm{O}}$ is considered as the reference point, and the chemical potential of gallium is then calculated based on the total energy of Ga$_2$O$_3$. On the other hand, in the Ga-rich condition, the system is assumed to have a high concentration of gallium compared to oxygen. The chemical potential of gallium for Ga-rich condition was taken from the total energy calculation of metallic gallium in its alpha phase. The chemical potential of Ir was determined from total energy calculations of ${\hbox {IrO}_{2}}$ and metallic Ir in the O-rich and Ga-rich limits, respectively.

### Estimation of correction energy

One of the challenges of the present work was to properly estimate the correction energy of charged defects $E_\text {corr}$, which is used to calculate the formation energy of the charged point defects.

The origin of the term $E_\text {corr}$ in Eq. (2) comes from using periodic boundary conditions. Clearly, the unit cell of the crystal cannot be charged, because in that case, the total energy would diverge. Because of that, any additional charge in the cell is compensated with a homogeneous background charge that ensures the neutrality of the cell. The correction energy accounts for the Coulomb interactions of the mirror images of the defects, as well as defect-background and background-background interactions.

To estimate the correction energy using CRYSTAL17 code^33^ we followed the methodology proposed in Ref.^47^. $E_\text {corr}$ consists of two parts. The first part, which is commonly is denoted as $\Delta V$, is the difference between the reference points of electrostatic potentials of neutral and charged systems. In non-periodic systems the reference point (or 0 value) is usually taken at the infinity distance, which is universal for all non-periodic systems. In periodic systems, however, there is no such point. Because of this we need to calculate a constant offset $\Delta V$ of the electrostatic potentials to compare the energies of two systems, namely, neutral and charged.

Following Ref.^47^, $\Delta V$ was calculated as a difference between the total energies of two systems: neutral defect-free system of infinitely large supercell and the same system, but without one electron. Of course, it is impossible to use the infinite supercell practically, instead, one should look at the tendency of $\Delta V$ with supercell size tending to infinity.

**Figure 4:**
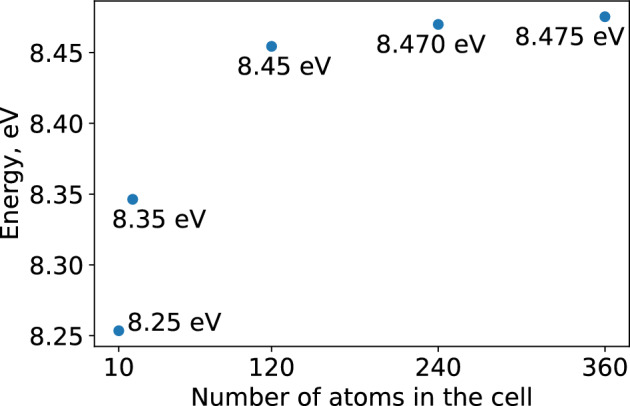
Energy difference between the $\beta$-$\hbox {Ga}_{2}\hbox {O}_{3}$ without one electron and ideal $\beta$-$\hbox {Ga}_{2}\hbox {O}_{3}$ at different supercell sizes with 10, 20, 120, 240 and 360 atoms.

As it can be seen from Fig. 4, we calculated the needed difference at 10, 20, 120, 240 and 360 atoms in the supercell for $\beta$-$\hbox {Ga}_{2}\hbox {O}_{3}$. The results for cells with 240 and 360 atoms differ by less than 0.01 eV. We assumed the value of $\Delta V$ to be 8.48 eV with a possible error of 0.01 eV. Similarly, we did the same calculations for $\alpha$-$\hbox {Ga}_{2}\hbox {O}_{3}$ and got the value of $\Delta V$ of 9.70 eV. The total energy of charged system should be corrected by this number times the charge of the unit cell $q \Delta V$.

The second part of the correction energy, which we denote as $\Delta E$, includes Coulomb interactions between charged point defect and it’s mirror images. Theoretically, it can be calculated using the formula^46^:

3 $$\begin{aligned} \Delta E = \frac{q^2 \alpha _M}{2 \varepsilon L} + O(L^{-3}) \end{aligned}$$

where *q* is the charge of the defect, $\alpha _M$ is the Madelung constant for the specific lattice, *L* is linear size of the cell and $\varepsilon$ is the dielectric constant. The value of $\frac{\alpha _M}{2L}$ can be directly computed with CRYSTAL code. In order to obtain it, we ran a calculation with the only atom in the supercell being the hydrogen atom located at a defect site. $\frac{\alpha _M}{2L}$ is the nucleus-nucleus interaction of the resulting system, which can be directly gathered from the calculation output. Dielectric constant $\varepsilon$ can be taken from literature or calculated directly with CRYSTAL17. In our work we took $\varepsilon$ from the literature ($\beta$-$\hbox {Ga}_{2}\hbox {O}_{3}$: $\varepsilon = 3.56$^48^, $\alpha$-$\hbox {Ga}_{2}\hbox {O}_{3}$: $\varepsilon = 3.75$^49^). With our supercell size (120 atoms, volume is around 1000 Å$^3$) $\Delta E$ turned out to be 0.5 eV.

## Conclusions

We calculated band structure and formation energies of iridium defects in $\beta$-$\hbox {Ga}_{2}\hbox {O}_{3}$ and $\alpha$-$\hbox {Ga}_{2}\hbox {O}_{3}$. In $\beta$-$\hbox {Ga}_{2}\hbox {O}_{3}$ octahedral site is highly more favorable for substitution than tetrahedral. From band structure calculations we predict electronic transitions with the energies of 0.5 and 3.0 eV, that most likely correspond to experimentally observed absorption energies at 5150 cm$^{-1}$ (0.64 eV) and 2.9 eV in $\beta$-$\hbox {Ga}_{2}\hbox {O}_{3}$. We justify that the origin of these energies indeed is the transitions between *d* orbitals in Ir$^{4+}$ substituting octahedrally coordinated gallium (Ga$_\text {II}$).

Defect formation energy diagram points to the fact that Iridium impurities in Czochralski grown $\beta$-$\hbox {Ga}_{2}\hbox {O}_{3}$ mono-crystals inhibit p-type conductivity. This prediction is in agreement with previously published studies^25^ and should be taken into account considering $\hbox {Ga}_{2}\hbox {O}_{3}$ as a p-type material (Supplementary File 1).

## Supplementary Information

Supplementary Information.

## Data Availability

The datasets used and analyzed during the current study are included in this published article in supplementary information as Supplementary File. “S1. Archive with input/output data of CRYSTAL code calculations”.
